# Blood Alcohol Concentration-Related Lower Performance in Immediate Visual Memory and Working Memory in Adolescent Binge Drinkers

**DOI:** 10.3389/fpsyg.2017.01720

**Published:** 2017-10-04

**Authors:** Concepción Vinader-Caerols, Aránzazu Duque, Adriana Montañés, Santiago Monleón

**Affiliations:** Department of Psychobiology, University of Valencia, Valencia, Spain

**Keywords:** blood alcohol concentration, binge drinking, immediate visual memory, working memory, adolescents

## Abstract

The binge drinking (BD) pattern of alcohol consumption is prevalent during adolescence, a period characterized by critical changes to the structural and functional development of brain areas related with memory and cognition. There is considerable evidence of the cognitive dysfunctions caused by the neurotoxic effects of BD in the not-yet-adult brain. Thus, the aim of the present study was to evaluate the effects of different blood alcohol concentrations (BAC) on memory during late adolescence (18–19 years old) in males and females with a history of BD. The sample consisted of 154 adolescents (67 males and 87 females) that were classified as refrainers if they had never previously drunk alcoholic drinks and as binge drinkers if they had drunk six or more standard drink units in a row for men or five or more for women at a minimum frequency of three occasions in a month, throughout the previous 12 months. After intake of a high acute dose of alcohol by binge drinkers or a control refreshment by refrainers and binge drinkers, subjects were distributed into four groups for each gender according to their BAC: BAC0-R (0 g/L, in refrainers), BAC0-BD (0 g/L, in binge drinkers), BAC1 (0.3 – 0.5 g/L, in binge drinkers) or BAC2 (0.54 – 1.1 g/L, in binge drinkers). The subjects’ immediate visual memory and working memory were then measured according to the Wechsler Memory Scale (WMS-III). The BAC1 group showed lower scores of immediate visual memory but not of working memory, while lower performance in both memories were found in the BAC2 group. Therefore, the brain of binge drinkers with moderate BAC could be employing compensatory mechanisms from additional brain areas to perform a working memory task adequately, but these resources would be undermined when BAC is higher (>0.5 g/L). No gender differences were found in BAC-related lower performance in immediate visual memory and working memory. In conclusion, immediate visual memory is more sensitive than working memory to the neurotoxic effects of alcohol in adolescent binge drinkers of both genders, being a BAC-related lower performance, and without obvious differences between males and females.

## Introduction

The binge drinking (BD) pattern of alcohol consumption is highly prevalent during adolescence. The quantity of alcohol, frequency of consumption and intermittency between binges have been shown to be important defining factors of BD and thus need to be delimitated in more detail. A blood alcohol concentration (BAC) of 0.8 g/L is required by BD criteria (National Institute of Alcohol Abuse and Alcoholism [NIAAA], 2004; Wechsler and Nelson, 2008), with men and women reaching this value after consuming 5 or more drinks and 4 or more drinks, respectively, in a short time period (2 h). This amount of alcohol is equivalent to the intake of approximately 60 g of alcohol in men and 50 g in women (6/5 or more drinks, respectively) (Parada et al., 2011a) when adapted to the Spanish population, although the Observatorio Español sobre Drogas [OED] (2016) accepts the criterion of 5/4 drinks (men/women respectively) in a 2-h period. A BD pattern is confirmed when frequency is at least once in the last 2 weeks (Courtney and Polich, 2009) or in the last month (Parada et al., 2011a), but using a longer time frame (last year) allows greater specificity in the classification of binge drinkers, as a necessary component of alcohol research and intervention (Cranford et al., 2006; Presley and Pimentel, 2006). Finally, the intermittence between BD episodes (according to the previously mentioned frequency) seems to be the most important factor involved, as the repeated alternation between intoxication and withdrawal is particularly deleterious for the brain, due to the excitotoxic cell death it provokes (Maurage et al., 2012; Petit et al., 2014).

Binge drinking is typically initiated during adolescence, a period (10–19 years old according to World Health Organization) characterized by critical changes to the structural and functional development of brain areas related with memory and cognition, particularly superior associative cortex (e.g., prefrontal cortex) which undergoes myelination, pruning and synaptic reorganization (Petit et al., 2013b; López-Caneda et al., 2014a), among other processes. Significant changes in the volume and shape of the hippocampal complex, another area which plays an important role in memory functions (e.g., immediate visual memory -IVM- and declarative memory), have also been observed in this developmental period (Gogtay et al., 2006; DeMaster et al., 2014; Krogsrud et al., 2014), being these changes remarkably heterogeneous among the different hippocampal subregions (Gogtay et al., 2006). In fact, alcohol-related performance deficits on tasks assessing cognitive processes such as attention, memory and executive functions, in the not-yet-adult brain are greater during adolescence (Crean et al., 2010; Risher et al., 2013) and become more pronounced with BD pattern consumption (López-Caneda et al., 2014a; Peeters et al., 2014). Thus, the BD adolescent population constitutes a risk cohort of brain damage, particularly if we bear in mind that it has been demonstrated that BD episodes can be more harmful for the brain than an equivalent amount of alcohol without withdrawal episodes (Duka et al., 2004; Petit et al., 2014).

Epidemiological studies have suggested that BD in youths is associated with an increased risk of alcohol abuse/dependence in adulthood (e.g., Chassin et al., 2002) or, in other words, that BD pattern may be considered as a precursor of alcohol use disorders (AUDs). In adolescents with AUDs, a reduction of hippocampal volume and prefrontal cortex has been observed (De Bellis et al., 2000, 2005), and has been related to cognitive deficits in IVM -more dependent of the hippocampus- and working memory (WM) -more dependent of the prefrontal cortex-.

In healthy late adolescents (up to 19 years old), the acute effects of alcohol on memory are poorly understood (Pihl et al., 2003). Despite the large amount of information provided by the literature, the majority of studies include broader age ranges and they encompass several developmental stages, such as youth (Grattan-Miscio and Vogel-Sprott, 2005; Schweizer et al., 2006; Day et al., 2013), adulthood (Dougherty et al., 2000; Weissenborn and Duka, 2003; Moulton et al., 2005; Söderlund et al., 2005; Paulus et al., 2006; Brumback et al., 2007; Rose and Duka, 2007; Saults et al., 2007; Cash et al., 2015), as well as older adults (Boha et al., 2009; Leitz et al., 2009; Bisby et al., 2010a,b; Montgomery et al., 2011; Poltavski et al., 2011; Wetherill and Fromme, 2011; McKinney et al., 2012; Hoffman and Nixon, 2015; Weafer et al., 2016). Besides the mnesic impact of acute BD, there are also studies showing effects of BD history in Spanish binge drinkers (Parada et al., 2011b; Sanhueza et al., 2011; Mota et al., 2013; Carbia et al., 2017) and international population (Squeglia et al., 2011; Campanella et al., 2013). On the other hand, research studying the effects of a BD episode’s BAC or other BACs on memory is inconsistent because of the influence of different factors, such as: a) the BD pattern has differential effects on the mnesic and executive functions dependent on the temporal-medial and prefrontal regions (López-Caneda et al., 2014a,b) the different types of memory are not similarly affected or are BAC-dependent (Sneider et al., 2013), and so there are studies showing a deterioration of visual memory (Schweizer et al., 2006) or the WM (Pihl et al., 2003; Grattan-Miscio and Vogel-Sprott, 2005; Schweizer et al., 2006; Saults et al., 2007; Day et al., 2013), while others do not observe any deleterious effect on the kind of memory in question (e.g., Moulton et al., 2005; Söderlund et al., 2005; Paulus et al., 2006; Rose and Duka, 2007); and c) the use of different memory tests (e.g., SOPT, Self-ordered Pointing Task; CANTAB, Cambridge Neuropsychological Test Automated Battery; BVRT, Benton’s Visual Retention Test…) for evaluating such mnesic effects.

Detrimental effects of alcohol use on cognitive functioning in adolescents are not limited to severe, long-term drinking behaviors and can be seen in dose-dependent episodic short-term drinking (Nguyen-Louie et al., 2015). Acute BD intoxication negatively affects spatial WM, planning abilities, response time and inhibition (e.g., Weissenborn and Duka, 2003; Squeglia et al., 2011; López-Caneda et al., 2014b). Evidence suggests that excessive drinking and resulting withdrawal symptoms dysregulate glutamine receptor activity, leading to degeneration and death of neurons. These sequelae of neurotoxic events may be detected through behavioral cognitive impairments in neuropsychological assessments (for reviews, see Jacobus and Tapert, 2013; López-Caneda et al., 2014a).

Gender differences in WM of young healthy subjects have been reported, indicating a male advantage in this memory, with females exhibiting disadvantages with a small effect size in both verbal and visuospatial WM (Zilles et al., 2016). This male advantage could be explained by activating effects of testosterone (Janowsky et al., 2000). Nevertheless, age and specific task modulate the magnitude and direction of the effects (e.g., Zilles et al., 2016; Voyer et al., 2017). This kind of differences is not so clear in IVM. Gender differences in the effects of alcohol have been also informed, supporting the view that the brains of male and female adolescents may be differentially affected by alcohol use (Alfonso-Loeches et al., 2013). There is evidence suggesting that female adolescents are more vulnerable to the neurotoxic effects of alcohol on cognition (Caldwell et al., 2005; Squeglia et al., 2011; Alfonso-Loeches et al., 2013), since the cognitive tolerance effect of alcohol on IVM develops in BD women but not in BD men (Vinader-Caerols et al., 2017). Other authors have found that men generally report lower sensitivity to alcohol (individuals need more alcohol to experience the same sensations or impairments) than women, and reactivity to alcohol-related cues is more pronounced in male than in female binge drinkers (e.g., Petit et al., 2013a). These results might at least partially explain why men typically show a higher prevalence of alcohol consumption than women. However, in Spain, the incidence of alcohol consumption in 14–18 year-old adolescents is higher in females than males (Observatorio Español sobre Drogas [OED], 2016). With respect to the BD pattern during adolescence, it is similar in 14–16 year-old adolescents and is more common among men than women in the age range of 17–18 years (Observatorio Español sobre Drogas [OED], 2016). A recent study in Spanish university alumni has revealed the existence of different typologies of alcohol users, with differences among males and females (Gómez et al., 2017). In the light of these data, it would seem crucial to consider gender differences when exploring the relationship between BD and memory in late adolescents.

Thus, considering (a) the different criteria that accompany the BD pattern initiated during the critical period of adolescence, (b) the unclear effects of alcohol, either acute consumption or BD history, on memory (IVM and WM), and (c) the potential greater vulnerability of women to the neurotoxic effects of alcohol; the aim of the present study was to evaluate the effects of different BACs on IVM and WM during late adolescence (18–19 years old) in healthy male and female individuals with a BD history (maintained during last year). We hypothesized a BAC-related lower performance on IVM and WM in adolescent binge drinkers, being women more sensitive than men. Also, having a BD history will be associated to lower performance compared to refrainers.

## Materials and Methods

### Participants

Experimental subjects were undergraduate students from the University of Valencia, Spain, who filled in a self-report questionnaire containing items enquiring about consumption of drugs, frequency and level of alcohol consumption, hours and quality of sleep, physical health, and psychological health. One hundred and fifty-four participants (67 males and 87 females, 18–19 years old) were recruited on the basis of the inclusion and exclusion criteria. The following inclusion criteria were used: 18–19 years old, a healthy body mass index (mean in men: 22.11 ± 0.34; and mean in women: 22.02 ± 0.31) and good health (without major medical problems). The exclusion criteria were as follows: taking medication; a history of mental disorders (diagnosed by a health professional according to DSM criteria); an irregular sleep pattern (non-restorative sleep and/or irregular schedule); having consumed, even sporadically, any drug (apart from alcohol) or having a history of substance abuse, including caffeine (our criterion: ≤2 stimulant drinks/day), tobacco (our criterion: ≤10 cigarettes/day), and alcohol; and having first-degree relatives with history of alcoholism. A telephone interview of approximately 15 min was conducted with each selected subject in order to confirm the information provided in the self-report and to arrange the date and time of the test.

Selected students were invited to participate in the study if they had reported refraining from alcohol consumption or a history of alcohol use classified as following a BD pattern according to the NIAAA criteria for Spain (see López-Caneda et al., 2014a) during the previous year. The mean age at onset of alcohol use was 14.7 ± 0.11 for binge drinkers. Participants were classified as refrainers if they had never previously drunk alcoholic drinks and as binge drinkers if they had drunk six or more standard drink units (SDU = 10 g of alcohol) in distilled spirits (alcohol content ≥40% vol., according to the BD habits referred by the subjects) in a row for men or five or more SDU in a row for women at a minimum frequency of three occasions in a month, throughout the previous 12 months.

Informed consent was obtained from all participants and the study was conducted in accordance with the guidelines for human experimentation of the Ethics Committee of the University of Valencia (ethical authorization number: H1380224121187) and with those of the Helsinki Declaration. Participants were told to abstain from drinking alcohol and performing heavy physical exercise during the evening/night prior to the experiment, and all subjects were instructed to follow their normal sleep patterns. Subjects were told to follow their usual meal routine at least 2 h before the experimental session.

The male subgroups consisted of 12–23 participants each, while the female subgroups consisted of 12–27 participants each. In the latter, data about the menstrual cycle were registered in the self-report and the telephone interview, and cycle phase was taken into account in the test in order to counterbalance this variable in each group.

### Tests and Apparatus

An alcoholmeter (Alcoquant^®^6020, Envitec, Germany) was employed to measure the BAC before and after intake of a drink.

The Alcohol Use Disorders Identification Test (AUDIT) (Saunders et al., 1993) was employed to measure alcohol abuse among the subjects. The AUDIT consists of 10 questions that evaluate the quantity and frequency of alcohol intake and alcohol-related behaviors and consequences. It uses a range of 0–40, in which a score of 8 or more in men and 6 or more in women indicates alcohol abuse. A higher score is related to greater severity of alcohol abuse.

The State-Trait Anxiety Inventory (STAI) (Spielberger, 1984) was used to measure anxiety. This is a questionnaire consisting of 20 items referring to self-reported state anxiety and 20 items referring to trait anxiety. All subjects completed the standardized Spanish version of the STAI.

IVM and WM were both assessed using the Wechsler Memory Scale 3rd Edition (WMS–III; adapted version for Spanish population) (Wechsler, 2004), a broadly used tool for assessing these kind of memories. The IVM subscale requires the respondent to recognize faces and remember scenes. The WM subscales require the respondent to put in order letter-number sets and reproduce visual-spatial sequences. Subjects’ scores on the IVM and WM scales were transformed into centiles according to the subject’s age.

### Procedure

All participants signed an informed consent and a confidentiality agreement of data on arrival at the laboratory. BAC was measured using the alcoholmeter in all subjects to ensure that they had not drunk alcohol previously on the day in question, and the alcohol use of the BD adolescent subjects was assessed using the AUDIT test (none of these subjects were assessed as alcohol-dependent). Then refrainers’ and binge drinkers’ drank 330 ml of lime- or orange-flavored refreshment (control groups) and binge drinkers’ drank a high acute dose of alcohol. Alcohol was administered in a fixed dose of 120 ml (38.4 g) consisting of vodka mixed with the refreshment for both genders or in function of their body weight (0.9 g alcohol / kg body weight in men and 0.8 g alcohol/kg body weight in women). The subjects were instructed to consume their drink within a period of 20 min. After finishing the drink, all subjects rinsed their mouths with water and BAC was repeatedly measured every 5 min throughout the waiting period, and until the BAC reached its peak (approximately 20 min after consuming the drink). This peak of BAC was considered the value to classify the participants into the experimental groups. The subjects performed then the STAI, IVM and WM tests. BAC was measured once again at the beginning of the tests, between the tests and at the end of the experiment. According to the BAC registered for the subjects (including the control groups, which consumed only the refreshment), four groups were constituted for each gender: BAC0-R (0 g/L, in refrainers), BAC0-BD (0 g/L, in binge drinkers), BAC1 (0.3 – 0.5 g/L, in binge drinkers) and BAC2 (0.54 – 1.1 g/L, in binge drinkers). Three subjects were excluded from the rest of the study because either they obtained a BAC under 0.3 g/L or they did not finish the drink.

All the tests were performed between 4:00 pm and 8:00 pm, and subjects that received alcohol remained on the premises until their alcohol concentration dropped to legal limits for driving (less than 0.3 g/L).

### Statistical Analyses

The data were subjected to parametric analysis after checking that they met the criteria for normality and homogeneity of variances. The BAC data for both genders were analyzed to ensure there were not statistically significant differences between the genders in the BAC1 and BAC2 groups. Next, an ANOVA was performed for each measure (IVM and WM); each analysis contained the between-subject factors “BAC” (BAC0-R, BAC0-BD, BAC1 and BAC2) and “Gender” (men and women) as independent variables. When their interaction was statistically significant, pairwise comparisons were carried out. The alpha values for comparisons were set at 0.049 and 0.0099, after applying the Bonferroni correction. All analyses were performed using the “SPSS” Statistics software package, version 22.0 for Windows (IBM Corp, 2013). Additionally, the statistical power of acute and chronic (BD history) effects of alcohol for IVM and WM was calculated by the G^∗^Power software, version 3.1.9.2 for Windows (the effect-size value was previously calculated using Cohen’s *d* formula).

## Results

The characteristics of the study population for refrainers and binge drinkers are summarized in **Table 1**.

**Table 1:** Characteristics of the study population.

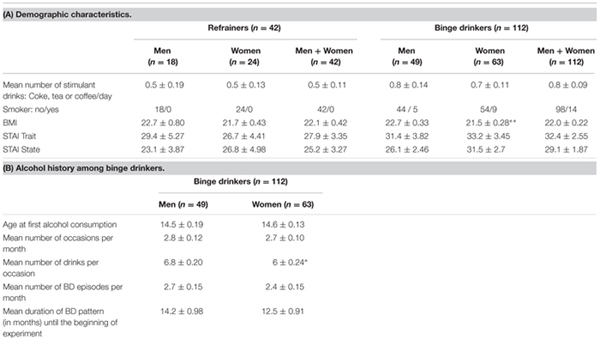

*The results are expressed as number or mean ± SEM for refrainers, occasional consumers, and binge drinkers. BMI, Body Mass Index. STAI, State and Trait Anxiety Inventory. ^∗^*p* < 0.05 Statistically significant difference between men and women in the same group according to Student’s *t*-tests. ^∗∗^*p* < 0.01 Statistically significant difference between men and women in the same group according to Student’s *t*-tests.*

**FIGURE 1:**
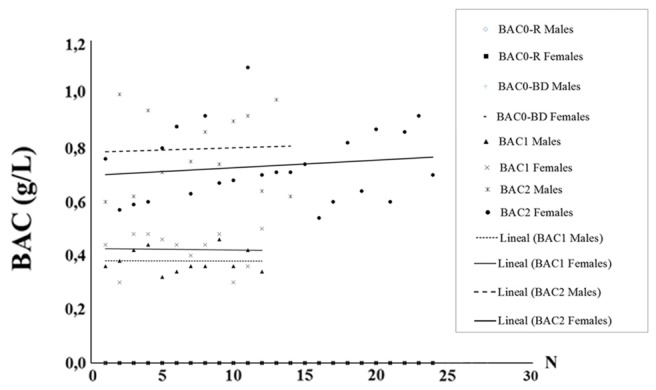
Scatterplot depicting the distribution of BACs obtained in the sample of male and female adolescents.

A scatterplot depicting the distribution of BACs in men and women is shown in **Figure 1**. ANOVA analyses did not show statistically significant differences between the genders in either BAC1 [F(1,22) = 3.472, *ns*] or BAC2 [F(1,36) = 0.304, *ns*].

A summary of descriptive statistics for IVM and WM is shown in **Table 2**.

**Table 2:** Descriptive statistics for IVM and WM.

	Immediate visual memory					Working memory				
		
	Men	*n*	Women	*n*	Statistical power^1^	Men	*n*	Women	*n*	Statistical power^1^
BAC0-R (0 g/L in refrainers)	39.24 ± 6.57	18	55.62 ± 5.49	24		56.34 ± 5.74	18	40.41 ± 4.05	24	
BAC0-BD (0 g/L in BD)	44.73 ± 5.65	23	40.51 ± 5.43	27	0.177	51.37 ± 4.99	23	36.05 ± 3.26	27	0.050
BAC1 (0.3-0.5 g/L)	23.89 ± 7.26	12	31.14 ± 10.17	12	0.987	43 ± 6.59	12	35.46 ± 6.58	12	0.386
BAC2 (0.54-1.1 g/L)	10.89 ± 4.49	14	26.68 ± 4.6	24	0.999	23.8 ± 4.4	14	23.55 ± 3.47	24	0.999


*The results are expressed as mean ± SEM. ^1^The Statistical Power is referred to comparisons with BAC0-R.*

### Immediate Visual Memory

**FIGURE 2:**
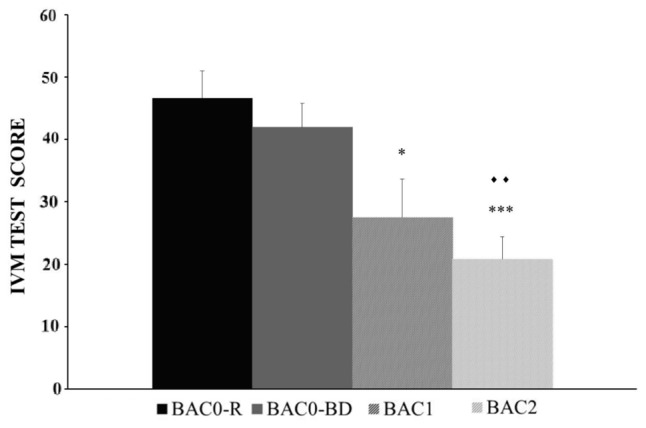
Performance of the Immediate Visual Memory task (Mean ± SEM) by male and female adolescents with different BACs (BAC0-R: 0 g/L, in refrainers; BAC0-BD: 0 g/L, in binge drinkers; BAC1: 0.3 – 0.5 g/L, in binge drinkers; and BAC2: 0.54 – 1.1 g/L, in binge drinkers). ^∗^*p* < 0.05, ^∗∗∗^*p* < 0.001 versus BAC0-R; 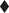 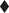 *p* < 0.005 *versus* BAC0-BD.

The BAC factor was statistically significant [*F*(3,146) = 9.354, *p* < 0.001], with a poorer performance of the IVM task registered in adolescents with BAC1 and BAC2 *versus* BAC0-R (*p* < 0.05 and *p* < 0.001, respectively), and in adolescents with BAC2 *versus* BAC0-BD (*p* < 0.005) (see **Figure 2**). In addition, no significant differences in performance were observed between BAC0-R and BAC0-BD groups (*p* > 0.05). Neither the Gender factor [*F*(1,146) = 3.847, *ns*] nor the BAC and Gender interaction [*F*(3,146) = 1.476, *ns*] was statistically significant.

### Working Memory

**FIGURE 3:**
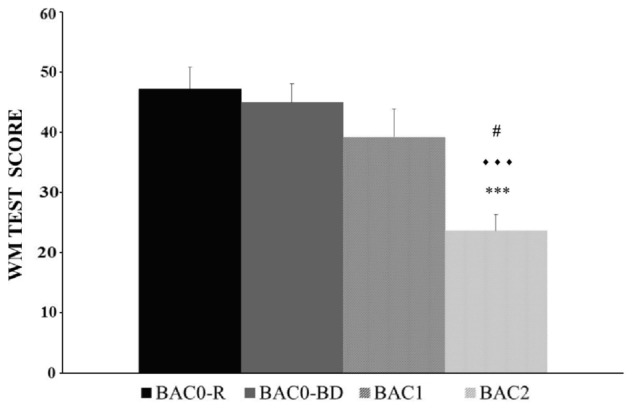
Performance of the Working Memory task (Mean ± SEM) by male and female adolescents with different BACs (BAC0-R: 0 g/L, in refrainers; BAC0-BD: 0 g/L, in binge drinkers; BAC1: 0.3 – 0.5 g/L, in binge drinkers; and BAC2: 0.54 – 1.1 g/L, in binge drinkers). ^∗∗∗^*p* < 0.001 versus BAC0-R; 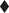 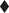 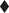 *p* < 0.001 versus BAC0-BD; # *p* < 0.05 *versus* BAC1.

**FIGURE 4:**
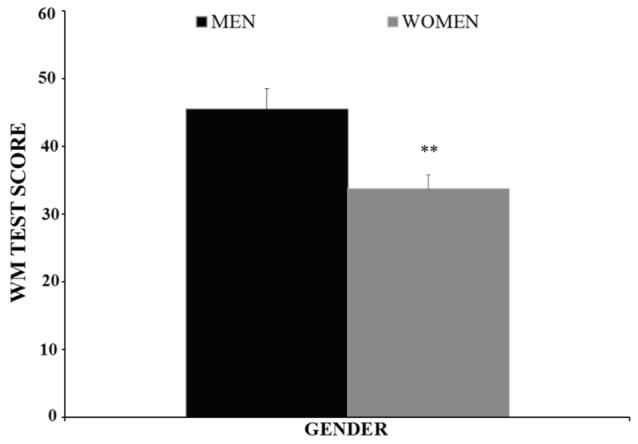
Performance of the Working Memory task (Mean ± SEM) by male and female adolescents. ^∗∗^*p* < 0.01 *versus* Men.

The BAC factor was statistically significant [*F*(3,146) = 10.353, *p* < 0.001]. The *post hoc* comparisons revealed that the adolescents in the BAC2 group (but not those in the BAC1 group) performed the WM task worse than those in the BAC0-R and BAC0-BD groups (*ps* < 0.001); BAC2 adolescents performed worse than their BAC1 counterparts (*p* < 0.05); and no significant differences were observed between the BAC0-R and BAC0-BD groups (*p* > 0.05); (see **Figure 3**). The factor Gender was also statistically significant [*F*(1,146) = 7.970, *p* < 0.01], with men performing better than women (see **Figure 4**). Finally, the interaction of BAC and Gender was not statistically significant [*F*(3,146) = 1.254, *ns*].

## Discussion

The main aim of the present study was to evaluate the neurotoxic effects of different BACs on IMV and WM in adolescent binge drinkers. To provide greater specificity in the classification of alcohol users among the university students that composed our study population, a moderate BAC (BAC1, around 0.4 g/L) and a BD BAC (BAC2, around 0.8 g/L) were evaluated in 18-19-year-old male and female binge drinkers that had met the criteria for a BD pattern over a longer time frame (during the previous year), with a minimum of three BD episodes per month. This pattern, characterized by repeated alternations between acute intoxication and withdrawal periods, is particularly neurotoxic, independently of the global alcohol intake (Maurage et al., 2012), as it leads to several cognitive impairments in the not-yet-adult brain (Jacobus and Tapert, 2013; López-Caneda et al., 2014a). One distinctive contribution of this work was to evaluate, together in the same study, the acute and chronic (BD history) impact of this pattern of alcohol consumption, as well as the possible gender differences.

In relation to IVM, our results show that the scores in this memory were lower in binge drinkers with a moderate BAC (BAC1, around 0,4 g/L) and a BD BAC (BAC2, around 0,8 g/L), whose performance was lower than that of refrainers. Nevertheless, binge drinkers with a moderate BAC, but not the BD BAC group, did not show any impairment of IVM with respect to binge drinkers that received the refreshment. A tolerance phenomenon could explain this lack of differences between these groups (BAC1 versus BAC0-BD), but the absence of a group of refrainers receiving alcohol in our design (for ethical reasons) does not allow us to directly evaluate this phenomenon. In a previous study (Vinader-Caerols et al., 2017), we observed an effect of tolerance on IVM impairment by alcohol in women but not in men. We are not aware of any study that has evaluated the effects of different BACs on IVM in adolescents, although damage to this memory has been reported with a BAC of 0.86 – 0.79 g/L (Schweizer et al., 2006), while other studies have not observed any deleterious effect with a similar BAC (Söderlund et al., 2005).

With respect to WM, the lower performance on this memory in the binge drinkers was dependent on BAC. Some authors have suggested that the lack of effect in tasks like WM could be due to that brain employs alternative networks, compensating the damage produced by alcohol (Tapert et al., 2001, 2004; Caldwell et al., 2005). Our results are in agreement with this interpretation suggesting that the brain of binge drinkers with a moderate BAC (around 0.4 g/L) could be employing compensatory mechanisms in additional brain areas to perform a WM task adequately (Tapert et al., 2004; Caldwell et al., 2005), but that these resources would be undermined when BAC is higher (>0.5 g/L). Such compensatory mechanisms have been reported in memories related to executive functions, as WM, in alcoholics (Desmond et al., 2003). These authors suggest that brain activation in left frontal and right cerebellar regions that control verbal WM may require a compensatory increase in order to maintain the same level of performance as controls. In the same way, previous studies have reported that young binge drinkers exhibit anomalies in neural activity involved in attentional/WM processes, and suggest that this anomalous neural activity reflects underlying dysfunctions in neurophysiological mechanisms, as well as the recruitment of additional attentional/WM resources to enable said binge drinkers to perform the task adequately (López-Caneda et al., 2013). Thus, our findings are in accordance with similar studies which found that acute alcohol (measured by breath alcohol content) was associated with an impairment of WM performance and mental flexibility, without affecting motor performance, measured by the Trail Making Test in 18–20 old adolescents with BD history (Day et al., 2013), and with reports of an impairment of WM with a BAC greater than 0.6 g/L (Pihl et al., 2003; Saults et al., 2007).

We cannot draw firm conclusions about the chronic effect of BD pattern on IVM and WM from our study. This is due to the lack of -for ethical reasons- a comparison group for the individuals that consumed a drink (i.e., a control group consisted of refrainers receiving a high dose of alcohol); as well as the low statistical power in the comparisons between binge drinkers that received refreshment and refrainers (see **Table 2**). Nevertheless, our results point out that maintaining a BD history over the previous year did not negatively affect the performance of the BAC0-BD group when compared with refrainers, either in IVM or WM. All the reviewed studies are in line with our results with respect to a BD history and IVM (e.g., García-Moreno et al., 2009; Parada et al., 2011b; Sanhueza et al., 2011; Mota et al., 2013). There are also several studies in agreement with our results in WM (e.g., Johnson et al., 2008; Winward et al., 2014; Boelema et al., 2015), where no differences in WM performance were observed between adolescents with BD history and controls. Similarly, Carbia et al. (2017) have reported that a stable BD during late adolescence and emerging adulthood is not associated with deficits in decision-making. Nevertheless, there are discrepancies amongst the literature, with studies showing better performance of refrainers in comparison to subjects with BD history (e.g., García-Moreno et al., 2008, 2009).

A greater cognitive vulnerability of women to the acute effects of alcohol has been highlighted by previous research (Vinader-Caerols et al., 2014, 2017); however, we have observed no gender differences in BAC-related lower performance in IVM and WM in the present study. We believe that an increased BAC cancels out these cognitive differences between men and women, though these interpretations obviously require further investigation. Independently of the BAC obtained, no genders differences were observed in IVM, but they were in WM, with men performing better than women. This is in accordance with other studies showing that visuospatial functioning of the WM is superior in males than in females (Rizk-Jackson et al., 2006; Vinader-Caerols et al., 2017). However, it should be mentioned that few studies have examined gender differences in WM and those that have done so report mixed results (Lejbak et al., 2011).

Our study suffers from some limitations which must be noted, such as the lack of an alcohol sensitivity measure (we will include an alcohol sensitivity questionnaire in our future research). Other variables apart from anxiety, such as depression or impulsivity, could have interfered with the interpretation of the results. Likewise, the use of different tests/batteries for evaluating IVM and WM (e.g., SOPT, Self-ordered Pointing Task; CANTAB, Cambridge Neuropsychological Test Automated Battery; BVRT, Benton’s Visual Retention Test…) contributes to the disparity of results from the studies in this field. Among these tasks, the Wechsler Memory Scale -employed in our study- is a broadly used tool for assessing this kind of memories. On the other hand, longitudinal studies that contemplate the moment of onset of adolescent BD would be useful in establishing the causes and effects of this pattern of alcohol use. Similarly, longitudinal studies could determine whether abnormalities in brain function persist or emerge if alcohol consumption is maintained (e.g., Correas et al., 2016), or whether they recover or brake their evolution when the binging ceases (e.g., López-Caneda et al., 2014b). Discovering the causes and effects of individual differences in alcohol consumption patterns is instrumental to designing programs and policy to reduce the impact of drinking in a highly vulnerable population such as adolescent and young people. Despite the mentioned limitations, the methodology of this work can provide unique empirical data on this field of research, taking into account the absence of research that focuses on the acute effects of alcohol in individuals younger than 20 years old.

## Conclusion

Our study shows that: (i) IVM is more sensitive than WM to the neurotoxic effects of acute alcohol in adolescents with a BD history, with BAC-related lower performance being noticeable (IVM score was lower with BAC1 and BAC2, while WM score was lower only when BAC reached levels of BD; i.e., around 0.8 g/L); and (ii) No gender differences are observed in BAC-related performance in IVM and WM (we believe that an increased BAC overrides these cognitive differences between men and women). Nevertheless, further research is needed in order to consolidate these conclusions.

## Author Contributions

CV-C and SM designed the study. AD and AM collected the data. CV-C, AD, and AM analyzed the data. CV-C and SM interpreted the data and wrote the first version of the manuscript. All authors collaborated on writing the final version of the manuscript.

## Conflict of Interest Statement

The authors declare that the research was conducted in the absence of any commercial or financial relationships that could be construed as a potential conflict of interest.
